# On Vaccine Inoculation

**Published:** 1806-04

**Authors:** 


### *To the Editors of the Medical and Physical Journal.*

Gentlemen,

THE extensive range of your valuable Journal, appears so appropriate for the communication and discussion of medical subjects, that I shall avail myself of the opportunity which it affords, to offer some remarks upon the cow-pox.

One would imagine that the weight of evidence, which has lately been brought forward from almost every quarter of the globe, in favour of Vaccine Inoculation, would have been sufficient to establish its fame and utility, and to silence the cavils of the sceptical and disputatious; but experience has not justified such expectations. Cases are perpetually quoted, which perplex the minds of practitioners, and disquiet the bosoms of anxious parents; a little serious reflection upon the subject, may possibly dissipate those fears, and strengthen the foundation upon which our assurances have been built, that the *true Cow-Pox* is, except in very few instances, an infallible preventive of infection of the small-pox, either by contagion or inoculation.

In order to maintain the truth of this proposition, it is necessary that every circumstance, through the several stages of the disease, should be carefully attended to, in such cases as are laid before the public, which has been, and I fear, generally speaking, ever will be, inclined to oppose whatever appears to be an *innovation* upon long established practice, not only in medical, but in every other science. That the judgment should pause, or pronounce with much caution, its approbation of any new mode of practice, is assuredly laudable; but when a mass of of evidence has been adduced, and innumerable proofs brought forward of the efficacy of the vaccine inoculation, and the numerous advantages attendant, surely all doubt should vanish from the mind. A mode of reasoning employed by a late philosophical writer, upon a different subject, appears not inapplicable to the present occasion.

"A wise man," says that acute reasoner, "proportions his belief to the evidence. In such conclusions as are founded upon an infallible experience, he expects the event with the last degree of assurance, and regards his past experience, as a full proof of the future existence of the event. In other cases he proceeds with more caution; he weighs the opposite experiments; he considers which side is supported by the greater number of experiments: to that side he inclines with doubt and hesitation; and when, at last, he fixes his judgment, the evidence exceeds not what some call *probability.* All probability, then, supposes an opposition of experiments and observations, when the one side is found to over-balance the other, and to produce a degree of evidence proportioned to the superiority. A hundred instances or experiments on one side, and fifty on another, afford a doubtful expectation of any event; though a hundred uniform experiments, with only one that is contradictory, reasonably beget a pretty strong degree of assurance. In all cases, we must balance the opposite experiments, where they are opposite, and deduct the smaller number from the greater, in order to know the exact force of the superior evidence."*

Though my experience in the cow-pox has not been very considerable, it has been sufficient to furnish me with some anomalous cases, and other practitioners have related to me a few more, which, had they been given to the public, in their crude state, might have occasioned much alarm. A more accurate investigation of the circumstances, has, however, cleared up the supposed failures, and accounted for the discordant phænomena. It could be wished that every person who vaccinated, whether gratuitously or otherwise, would keep a register of the name, age, place of abode, and general state of the health of each patient; examine the pustule from the third to the tenth day of vaccination; examine the body also, to discover, whether it had been subject to, or was then affected with herpetic or other eruptions of the skin, and enquire, likewise,

* See Hume's Essays, vol. II. p. 119.

likewise, if the patient had ever had the chicken-pox, a disease nearly resembling, and not unfrequently mistaken for small-pox.

If these circumstances were attended to, and noted accordingly, it would be very easy, after recording the event of such case, to refer to their memoranda; and if the usual symptoms had been of a suspicious or doubtful nature, it could not be esteemed extraordinary, that the patient should afterwards become infected with the small-pox, either by the lungs, or the lancet. It is now some time since the nobility and gentry of Sussex, under the patronage of the Prince of Wales, liberally came forward, to establish an institution for the extirpation of the small-pox. Many similar institutions have been proposed and established by disinterested professional gentlemen; and lately at Nottingham, as your useful Journal informs us. Such examples are highly worthy imitation, and would probably be universally followed, if every medical practitioner would agree with his brother, within certain districts, not to inoculate the small-pox. Such provincial institutions would, I presume, in the course of a few years totally annihilate this destructive poison. But without some general concurrence of this kind, it is in vain, I fear, to expect those beneficial and extensive advantages, which might be derived from vaccine inoculation. It is a melancholy fact, that prejudice exerts its baneful influence, principally among the uninformed and lower ranks of society, though the higher orders, it is to be deplored, are not totally free from it. To remove, if possible, this rooted prejudice, let us apply to both ranks, the most convincing of all kinds of evidence, (viz.) the evidence of the senses. Let those who have undergone the *true Vaccine Disease*, be inoculated for the small-pox, in the presence of a few persons in each district, who disbelieved the prophylactic power of cow-pox, and the result could not fail to produce the desired conviction. If these or similar means, suggested by others, should be found unsuccessful, we must trust to *Time*, which we have known to establish many truths, which, at their first promulgation, the most powerful arguments could not defend or support, and rest assured that the obstacles thrown in the way of this most happy and fortunate discovery, will ultimately rather increase than diminish its value and its importance.

I am, &c.

*March* 7, 1806. VIGORNIENSIS.

